# Monotherapy in patients with pulmonary arterial hypertension at four German PH centres

**DOI:** 10.1186/s12890-021-01499-2

**Published:** 2021-04-21

**Authors:** Beate Stubbe, Hans-Jürgen Seyfarth, Janina Kleymann, Michael Halank, Hussam Al Ghorani, Anne Obst, Susanna Desole, Ralf Ewert, Christian F. Opitz

**Affiliations:** 1 Internal Medicine B, Pneumology, University Hospital Greifswald, Greifswald, Germany; 2 Internal Medicine, Pneumology, University Hospital Leipzig, Leipzig, Germany; 3 Internal Medicine, Pneumology, University Hospital Dresden, Dresden, Germany; 4 Internal Medicine, Cardiology, DRK Kliniken Berlin and Charité-Universitätsmedizin Berlin, Berlin, Germany

**Keywords:** Pulmonary arterial hypertension (PAH), Monotherapy, Combination therapy, Comorbidities, Survival

## Abstract

**Background:**

Although combination therapy is the gold standard for patients with pulmonary arterial hypertension (PAH), some of these patients are still being treated with monotherapy.

**Methods:**

We conducted a retrospective analysis at four German PH centres to describe the prevalence and characteristics of patients receiving monotherapy.

**Results:**

We identified 131 incident PAH patients, with a mean age of 64 ± 13.8 years and a varying prevalence of comorbidities, cardiovascular risk factors and targeted therapy. As in other studies, the extent of prescribed PAH therapy varied with age and coexisting diseases, and younger, so-called “typical” PAH patients were more commonly treated early with combination therapy (48% at 4–8 months). In contrast, patients with multiple comorbidities or cardiovascular risk factors were more often treated with monotherapy (69% at 4–8 months). Survival at 12 months was not significantly associated with the number of PAH drugs used (single, dual, triple therapy) and was not different between “atypical” and “typical” PAH patients (89% vs. 85%).

**Conclusion:**

Although “atypical” PAH patients with comorbidities or a more advanced age are less aggressively treated with respect to combination therapy, the outcome of monotherapy in these patients appears to be comparable to that of dual or triple therapy in “typical” PAH patients.

## Background

The current international [1] and national (Cologne Consensus Conference, [2]) Guidelines for the diagnosis and treatment of P(A)H provide a comprehensive overview of supportive, targeted and interventional therapeutic options. It is recommended that targeted PAH therapy be implemented according to the risk profile. For this reason, various findings and parameters are used to categorize patients into three risk groups, consisting of low, intermediate and high estimated one-year mortality. Nevertheless, early combination therapy is the gold standard for most patients with PAH [3], and several meta-analyses support this approach [4–8]. However, the patients included in these studies do not necessarily represent the entire spectrum of patients routinely treated at PH centres and described in PAH registries. For example, patients included in randomized controlled trials tend to be younger and have fewer comorbidities and cardiovascular risk factors. Comparative studies of these different patient groups indicate that PAH combination therapy in elderly patients with multiple cardiovascular risk factors (so-called “atypical” PAH patients) may be associated with a higher rate of side effects and reduced efficacy [9]. A post hoc analysis of the AMBITION trial confirmed these findings [10]. These data have been considered in the German recommendations (Cologne Consensus Conference, [2]. Hence, these “atypical” patients, when assigned to the low- or intermediate-risk group, might be treated with monotherapy [11]. It remains to be seen whether such an approach will generally be adopted. Accordingly, initial monotherapy was also mentioned in the 6^th^ world symposium as an appropriate treatment option for selected patients [3]. These include older PAH patients (> 75 years) with cardiovascular risk factors for the presence of heart failure with preserved ejection fraction and patients with portopulmonary hypertension or uncorrected congenital heart defects.

The presented analysis aimed to answer the following questions: (1) what is the proportion of PAH patients treated with monotherapy in daily routine at German PH centres; (2) do PAH patients with comorbidities receive monotherapy more frequently; and (3) do PAH patients receiving monotherapy have poorer outcomes?

## Methods

### Patients

Out of 782 PH patients treated at four German PH centres between 2016 and 2018, 158 were classified as having PAH. In this group, complete data, including data on comorbidities, cardiovascular risk factors and PAH medications, were available for 131 incident PAH patients, representing the group analysed.

Patients were categorized as having “typical” or “atypical” PAH according to the criteria proposed by the Cologne Consensus Conference [11]. “Atypical” patients were defined as being > 65 years old and having ≥ 3 of the following comorbidities or cardiovascular risk factors: arterial hypertension, coronary heart disease, diabetes mellitus, obesity (BMI > 30 kg/m^2^), diastolic dysfunction (by echocardiography) or atrial fibrillation (Table 1).

**Table 1:** Characteristics of PAH patients (n = 131)

	Total n = 131	Typical patients N = 86	Atypical patients N = 45	*p* value
Age (years)	64 (± 13.8)	59 (± 14.1)	74 (± 5.3)	< 0.001
Male	65 (49.6%)	48 (55.8%)	17 (37.8%)	< 0.050
BMI (kg/m^2^)	28.1 (± 6.4)	27.8 (± 7.0)	28.9 (± 5.0)	0.296
Diagnoses				
IPAH	48 (36.6%)	28 (32.6%)	20 (44.4%)	0.180
PAH	83 (63.4%)	58 (67.4%)	25 (55.6%)	
Portopulmonary PAH	11 (8.4%)	10 (17.2%)	1 (4.0%)	
PAH due to connective tissue disease	40 (30.5%)	23 (39.7%)	17 (68.0%)	
PAH due to congenital heart disease	3 (2.3%)	3 (5.2%)	-	
*Further diagnoses*				
Cardiovascular disease				
Diastolic dysfunction (by echocardiography)	62 (47.3%)	30 (34.9%)	32 (71.1%)	< 0.001
Arterial hypertension	95 (72.5%)	54 (62.8%)	41 (91.1%)	< 0.001
Coronary heart disease	32 (24.4%)	14 (16.3%)	18 (40.0%)	0.003
Atrial fibrillation	37 (28.4%)	12 (14.0%)	25 (55.6%)	< 0.001
Peripheral arterial occlusive disease	6 (4.6%)	3 (3.5%)	3 (6.7%)	0.409
Thromboembolic disease	19 (14.5%)	9 (10.5%)	10 (22.2%)	0.070
Pulmonary embolism	12 (63.2%)	6 (66.6%)	6 (60.0%)	
Diabetes mellitus	38 (29.0%)	15 (17.4%)	23 (51.1%)	< 0.001
Insulin dependence	17 (44.7%)	6 (40.0%)	11 (47.8%)	
Chronic kidney disease	64 (48.9%)	32 (37.2%)	32 (71.1%)	< 0.001
Thyroid disease	30 (22.9%)	17 (19.8%)	13 (28.9%)	0.238
Pulmonary disease				
COPD	30 (22.9%)	21 (24.4%)	9 (20.0%)	0.568
ILD	21 (16.0%)	13 (15.1%)	8 (17.8%)	0.693
Cancer	11 (8.4%)	5 (5.8%)	6 (13.3%)	0.186
Obstructive sleep apnoea	12 (9.2%)	7 (8.1%)	5 (3.8%)	0.751
Functional class				
II	18 (13.7%)	15 (17.4%)	3 (6.7%)	0.107
III	90 (68.7%)	54 (62.8%)	36 (80.0%)	
IV	23 (17.6%)	17 (19.8%)	6 (13.3%)	
6-MWD (m) (n = 57)	266 (± 129)	266 (± 139) n = 38	267 (± 107) n = 19	0.959
*Pulmonary function*				
FVC %pred	83.4 (± 20.9) (n = 93)	82.8 (± 20.4) (n = 65)	84.7 (± 22.2) (n = 28)	0.696
FVC %pred < 70%	21 (22.6%)	14 (21.5%)	7 (25.0%)	
FEV_1_%pred	76.7 (± 19.9) (n = 94)	75.3 (± 19.1) (n = 66)	80.1 (± 21.6) (n = 28)	0.284
FEV_1_%pred < 60%	21 (22.3%)	16 (24.2%)	5 (17.9%)	
FEV_1_/FVC	74.4 (± 12.4) (n = 94)	74.2 (± 13.4) (n = 66)	75.0 (± 9.9) (n = 28)	0.772
FEV_1_/FVC < 70%	31 (33.0%)	24 (36.4%)	7 (25.0%)	
DLCO %pred	41.5 (± 17.2) (n = 82)	42.1 (± 19.0) (n = 59)	39.9 (± 11.9) (n = 23)	0.534
DLCO %pred < 45%	49 (59.8%)	34 (57.6%)	15 (65.2%)	
paO_2_ (mmHg)	62.0 (± 17.8) (n = 93)	63.8 (± 17.1) (n = 65)	57.8 (± 18.9) (n = 28)	0.138
paCO_2_ (mmHg)	33.5 (± 6.8) (n = 93)	33.2 (± 6.0) (n = 65)	34.2 (± 8.6) (n = 28)	0.571
CPET				
VO_2_ peak %pred	49.9 (± 16.1) (n = 73)	50.0 (± 16.7) (n = 48)	49.6 (± 15.2) (n = 25)	0.917
VO_2_ peak (ml/kg/min)	11.8 (± 4.2) (n = 73)	12.6 (± 4.6) (n = 48)	10.1 (± 2.7) (n = 25)	0.004
VE/VCO_2_ slope	52.7 (± 17.8) (n = 71)	51.4 (± 17.6) (n = 48)	55.3 (± 18.3) (n = 23)	0.396
Haemodynamics				
PAPm (mmHg)	48 (± 12.8)	48.9 (± 13.7) (n = 86)	45.9 (± 10.5) (n = 45)	0.197
RAPm (mmHg)	10 (± 5.1) (n = 126)	9.5 (± 5.4) (n = 82)	10.3 (± 4.5) (n = 44)	0.416
PAOPm (mmHg)	12 (± 4.9) (n = 126)	11.5 (± 4.6) (n = 84)	12.7 (± 4.3) (n = 42)	0.162
CI (l/min/m^2^)	2.4 (± 0.8) (n = 115)	2.4 (± 0.9) (n = 76)	2.4 (± 0.7) (n = 39)	0.701
PVR (Wood Unit)	9.2 (± 4.9) (n = 119)	9.7 (± 5.4) (n = 78)	8.4 (± 3.7) (n = 41)	0.156
Echocardiography				
TAPSE (mm)	18 (± 5.3) (n = 100)	17.4 (± 4.8) (n = 63)	17.6 (± 6.2) (n = 37)	0.893
PAP syst. (mmHg)	72 (± 21.9) (n = 104)	77.8 (± 23.8) (n = 62)	74.9 (± 18.9) (n = 42)	0.502
RA surface (cm^2^)	27 (± 6.8) (n = 82)	26.2 (± 6.2) (n = 50)	27.9 (± 7.7) (n = 32)	0.256
Pericardial effusion	89	60	29	
Yes	12 (13.5%)	9 (15.0%)	3 (10.3%)	

Continuous data are expressed as the mean (± std); nominal data are given as values and percentages

*BMI* body mass index, *COPD* chronic obstructive pulmonary disease, *ILD* interstitial lung disease, *6-MWD* 6-min walking distance

*p values for comparison of typical versus atypical patients

In addition, a broad spectrum of comorbidities potentially affecting the outcome in these patients were recorded, including chronic kidney disease, thromboembolic disease, peripheral arterial occlusive disease, chronic obstructive pulmonary disease (COPD), interstitial lung disease (ILD), cancer and obstructive sleep apnoea.

### Data collection

The four contributing university PH centres are considered representative of German PH centres, as they are well-established institutions with documented expertise in diagnosing and treating PH patients. Furthermore, they regularly participate in clinical studies and maintain good collaboration. The number of PH patients treated with monotherapy at these four centres is comparable to data on the German registry (COMPERA registry, data on file).

The following data were collected retrospectively from medical records: age, sex, weight, height, secondary diagnoses, selected echocardiographic parameters, spiroergometric parameters, 6-min walking distance and haemodynamic parameters. The prescribed PAH drugs were documented for the entire observation period, and the vital status (alive, dead, transplanted, lost to follow-up) was recorded at the end of follow-up on September 30, 2019.

### Follow-up

Follow-up data were collected at 0—3 months (baseline), 4—8 months (1st follow-up) and 9—15 months (2nd follow-up).

### Statistics

Continuous data are presented as the mean (± standard deviation), and categorical variables are presented as absolute frequencies and percentages. The t-test was used to compare selected parameters between patients with “typical” or “atypical” PAH. Categorical variables were compared by the chi-square test, Fisher’s exact test, or the McNemar test. Survival was evaluated using Kaplan–Meier analysis, and differences between groups were assessed by the log-rank test. Analyses were performed with SAS 9.4 (SAS Institute, Inc., Cary, NC, USA).

The study was approved by the ethics committee of the University of Greifswald (Reg. No. BB 167/18, with an amendment to extend the observation period).

## Results

The study included 131 patients (49.6% male), of whom 48 (36.6%) were classified as having idiopathic pulmonary arterial hypertension (IPAH) and 83 (63.4%) were classified as having pulmonary arterial hypertension (PAH). At baseline, the mean age was 64 ± 13.8 years, and the functional class (FC) was III in 90 (68.7%) and IV in 23 patients (17.6%) (Table 1). The average 6-min walking distance was 266 ± 129 m.

Considering cardiovascular risk factors and age, 86 (65.6%) patients were classified as having “typical” PAH and 45 (34.4%) were classified as having “atypical” PAH. Comparing “typical” and “atypical” PAH patients, significant differences between the two groups were found in terms of age, sex, diastolic dysfunction, arterial hypertension, coronary heart disease, atrial fibrillation, diabetes mellitus, chronic kidney disease and peak oxygen uptake (Table 1).

**Fig. 1:**
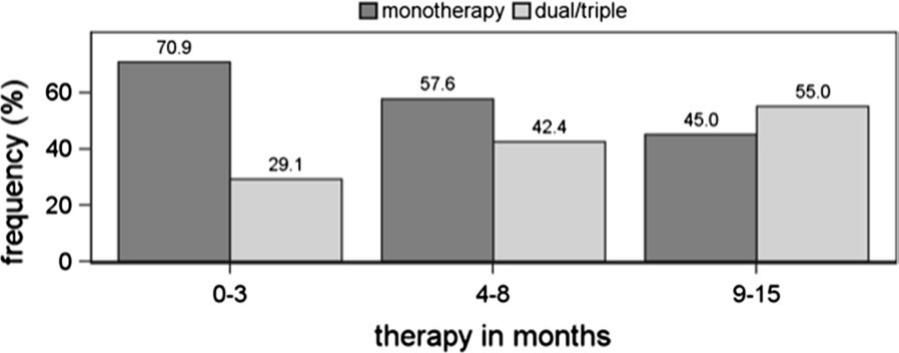
Duration of targeted treatment

At baseline, 117/131 (89.3%) patients were treated with targeted PAH therapy, with 83 (70.9%) receiving monotherapy, 27 (23.1%) receiving dual therapy and 7 (6.0%) receiving triple therapy. At the first follow-up, 125/131 patients (95%) were treated with PAH therapy, of whom 72 (57.6%) continued to receive monotherapy, while 53 (42.4%) were on dual or triple therapy. At the second follow-up, 111/131 patients (85%) were available, of whom 50 (45.0%) continued to receive monotherapy, while 61 (55.0%) were on dual or triple therapy (Fig. 1). Overall, the median follow-up duration was 22 months (13; 30).

Regarding the “atypical” or “typical” phenotype, we found significant differences in the distribution of monotherapy vs. dual/triple therapy at baseline (*p* = 0.036), a pattern that persisted, although no longer significant, at the first and second follow-ups (Table 2). In “atypical” patients, we observed an increased proportion of combination therapy over time (baseline to first follow-up, *p* = 0.014 and to second follow-up, *p* = 0.002).

**Table 2:** Targeted PAH therapy in patients

Baseline	Typical	Atypical	*p* value
PAH therapy, 0–3 months	n = 86	n = 45	
None	10 (11.6%)	4 (8.9%)	0.212
Monotherapy	49 (57.0%)	34 (75.6%)	
Dual therapy	21 (24.4%)	6 (13.3%)	
Triple therapy	6 (7.0%)	1 (2.2%)	
	n = 76	n = 41	
Monotherapy	49 (64.5%)	34 (82.9%)	0.036
Dual and triple therapy	27 (35.5%)	7 (17.1%)	
PAH therapy, 4–8 months	n = 83	n = 42	
Monotherapy	43 (51.8%)	29 (69.0%)	0.119
Dual therapy	33 (39.8%)	9 (21.4%)	
Triple therapy	7 (8.4%)	4 (9.5%)	
	n = 83	n = 42	
Monotherapy	43 (51.8%)	29 (69.0%)	0.065
Dual and triple therapy	40 (48.2%)	13 (31.0%)	
PAH therapy, 9–15 months	n = 72	n = 39	
Monotherapy	30 (41.7%)	20 (51.3%)	0.576
Dual therapy	35 (48.6%)	15 (38.5%)	
Triple therapy	7 (9.7%)	4 (10.3%)	
	n = 72	n = 39	
Monotherapy	30 (41.7%)	20 (51.3%)	0.331
Dual and triple therapy	42 (58.3%)	19 (48.7%)	

Data are given as percentages

With increasing age, the proportion of patients treated with combination therapy decreased. At baseline, older patients received more monotherapy (*p* = 0.016). This difference was no longer significant at the first or second follow-up (Table 3). However, in patients over 65 years of age, we observed an increased proportion of combination therapy over time (baseline to first follow-up, *p* < 0.001 and to second follow-up, *p* < 0.001).

**Table 3:** Dependence of targeted PAH therapy on age

Baseline	≤ 65 years	> 65 years	*p* value
PAH therapy, 0–3 months	n = 59	n = 72	
None	7 (11.9%)	7 (9.7%)	0.046
Monotherapy	31 (52.5)	52 (72.2%)	
Dual therapy	15 (25.4%)	12 (16.7%)	
Triple therapy	6 (10.2%)	1 (1.4%)	
	n = 52	n = 65	
Monotherapy	31 (59.6%)	52 (80.0%)	0.016
Dual and triple therapy	21 (40.4%)	13 (20.0%)	
PAH therapy, 4–8 months	n = 57	n = 68	
Monotherapy	28 (49.1%)	44 (64.7%)	0.171
Dual therapy	22 (38.6%)	20 (29.4%)	
Triple therapy	7 (12.3%)	4 (5.9%)	
	n = 57	n = 68	
Monotherapy	28 (49.1%)	44 (64.7%)	0.079
Dual and triple therapy	29 (50.9%)	24 (35.3%)	
PAH therapy, 9–15 months	n = 48	n = 63	
Monotherapy	19 (39.6%)	31 (49.2%)	0.296
Dual therapy	22 (45.8%)	28 (44.4%)	
Triple therapy	7 (14.6%)	4 (6.3%)	
	n = 48	n = 63	
Monotherapy	19 (39.6%)	31 (49.2%)	0.313
Dual and triple therapy	31 (60.4%)	32 (50.8%)	

Data are given as percentages

### Survival

**Fig. 2:**
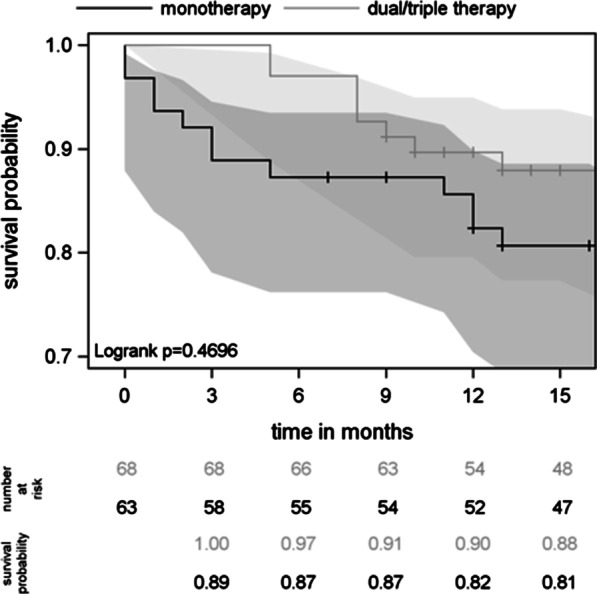
Survival curves under PAH treatment with confidence intervals

**Fig. 3:**
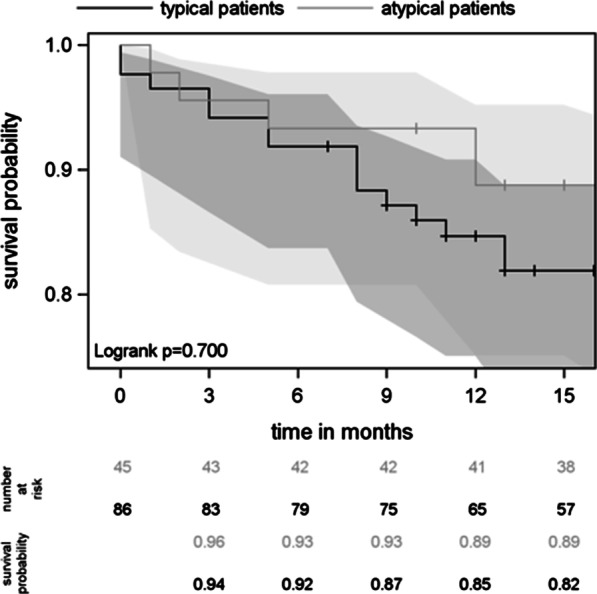
Survival curves of typical and atypical patients with confidence intervals

**Fig. 4:**
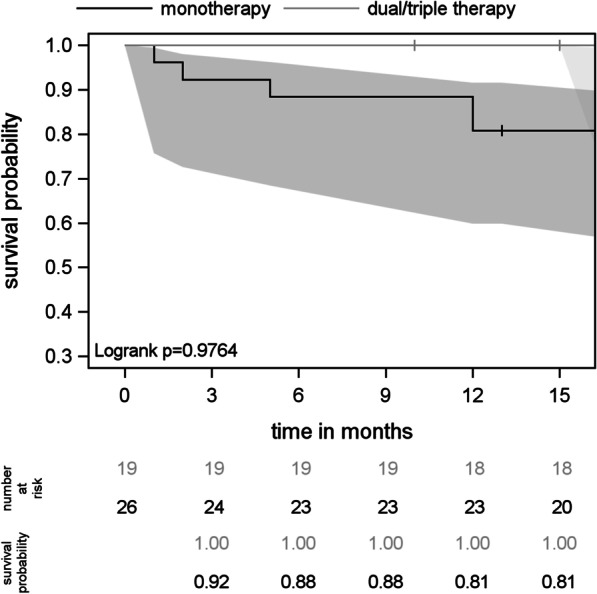
Survival curves in atypical PAH patients on monotherapy versus combination therapy

There was no significant difference (*p* = 0.411) in survival with respect to the number of PAH drugs prescribed (Fig. 2). Accordingly, at 12 months, survival was similar between patients with “atypical” and “typical” PAH (89% vs. 85%, *p* = 0.700, Fig. 3). Within the “atypical” PAH group (N = 45), survival at 12 months did not differ between patients on combination therapy and those on monotherapy (Fig. 4).

## Discussion

This study enrolled 131 incident PAH patients treated at four German PH centres between 2016 and 2018. The mean patient age of 64 ± 13.8 years is comparable to that of patients in registry studies used for risk assessment [12–14] but higher than that in recently published clinical trials [15–18]. Among recent randomized clinical trials, the mean age was 54 ± 14 years in the AMBITION trial [19], 46 ± 16 years in the SERAPHIN trial [20] and 48 ± 15 years in the GRIPHON trial [21].

Only 36.6% of our patients were classified as having IPAH; however, this number reached up to 75% in clinical trials [12] and ranged between 46 and 63% in PH registries [22, 23]. One reason for this difference might be the high proportion of PAH patients with comorbidities or cardiovascular risk factors. In previous registry studies, such data were not systematically collected [24–26]. The English ASPIRE registry reported comorbidities in 37% of their CTEPH patients [27]. For the first time, a more complete analysis of comorbidities and cardiovascular risk factors was performed in the American REVEAL registry [23]. In contrast, the COMPERA registry [28] obtained data for only a limited number of comorbidities and cardiovascular risk factors, although these investigators pointed out a significant increase in the age of their newly diagnosed IPAH patients. In later studies [19, 29] as well as registry analyses [13, 30], these data were documented more comprehensively. Remarkably, the amendment redefining the eligibility criteria in the recruiting phase of the AMBITION trial to implement more stringent haemodynamic requirements and exclude patients with ≥ 3 risk factors for left ventricular diastolic dysfunction led to a change in the study population [19]. The background of this modification was based on the observation that a relevant proportion of the initially recruited patients had cardiovascular risk factors (BMI ≥ 30 kg/m^2^, arterial hypertension, diabetes mellitus, relevant coronary heart disease). This subgroup of patients was described as having “atypical” PAH to distinguish them from “classical” IPAH patients with few comorbidities [31]. This terminology was adopted in subsequent studies [9] and in the German recommendations for the diagnosis and treatment of PH. Certain comorbidities and cardiovascular risk factors are more common in our patients than in other cohorts, especially arterial hypertension (Table 4). It remains unclear whether these differences in risk factor and comorbidity profiles are due to variations in data acquisition or represent distinct patient populations [32]. The incidence of echocardiographic signs of heart failure with preserved ejection fraction, which is not even reported in most studies or registries, could be documented in almost 50% of our patients, although not all criteria of the most recent definition of “heart failure with preserved ejection fraction” were met [33]. Our findings are in line with those of previous reports describing frequent signs of “left ventricular diastolic dysfunction” in patients with IPAH [34]. Among the other comorbidities, both chronic kidney disease [13, 35] and ischaemic heart disease [13] are prognostically relevant. For this reason, chronic kidney disease is part of the REVEAL risk score [36]. Pulmonary hypertension is a frequent finding in patients with chronic kidney disease [37, 38]. Moreover, with increasing age, the incidence of kidney dysfunction increases in patients with PAH (63% for IPAH patients 65–74 years old, 85% for those ≥ 75 years old) [13]. It is not yet clear whether patients with PH and kidney dysfunction should be categorized in WHO Group V or classified as PAH patients with renal comorbidity [39].

**Table 4:** Comparison of comorbidities in selected studies

Comorbidity	Current study	Study 1 (12)	Study 2 (30)	Study 3 (23)
No. of patients	131	264	237	1247
BMI (kg/m^2^)	28.1 ± 6.4	27.5 ± 5.5	29.6 ± 8.3	n.a
Arterial hypertension	72.5	51	30	38.9
Diabetes mellitus	29	29	17.7	10.2
Ischaemic stroke	n.a	7	n.a	n.a
Ischaemic heart disease	24.4	18	40.9	8.9
Atrial fibrillation	28.4	17	n.a	n.a
Obesity	29.8	21	n.a	28.6
Chronic kidney disease	48.9	51	15.6	4.8
COPD	22.9	n.a	19.8	21.1
OSA	9.2	n.a	19	15.9
Depression	n.a	n.a	n.a	25.3
Thrombosis, pulmonary embolism	14.5	n.a	n.a	11.9
Diastolic dysfunction by echocardiography	47.3	n.a	n.a	n.a
Thyroid disease	22.9	n.a	n.a	23.2
Cancer	8.4	n.a	n.a	5.9
Peripheral arterial occlusive disease	4.6	n.a	n.a	n.a

Continuous data are expressed as the mean (± SD); nominal data are given as percentages

Similar to other chronic diseases, such as chronic heart failure [40] or COPD [41], the prevalence of comorbidities increases with age and affects survival in PAH patients. Therefore, the treatment of these comorbidities can also improve the prognosis of the “primary” disease, in this case, PAH [42]. On the other hand, previous studies have suggested that the clinical response to targeted PAH drugs can be comparable, irrespective of the number of comorbidities [9, 10, 43]. Recent studies using cluster analyses have described different IPAH phenotypes based on age, the presence of cardiovascular risk factors and comorbidities and selected echocardiographic, spiroergometric and haemodynamic findings [44], as done previously in patients with pulmonary heart disease [45]. These data suggest that so-called type II pulmonary heart disease, with severe pulmonary vascular involvement and right ventricular dysfunction, is comparable to PAH. A similar approach (cluster analysis) was performed on IPAH patients in the COMPERA registry, linking different phenotypes with survival [46]. It remains to be seen whether such phenotype classifications will affect therapeutic strategies for PAH patients in the future, as has been proposed for other disease entities, such as heart failure with preserved ejection fraction [47]. In the recently published COMPERA cluster analysis [46] of 846 IPAH patients, 38% and 63% of “typical” patients (median age of 45 years old, without so-called “risk factors for left heart disease”) were treated with combined targeted PAH therapy within the first three months and after one year during follow-up, respectively. The other patients were predominantly treated with monotherapy at baseline and during follow-up.

It remains an important goal to treat PAH patients according to the current guidelines and reduce the gap between patients who do and do not receive aggressive combination therapy, when appropriate [48, 49]. Accordingly, recent data indicate that the use of combination therapy in patients with PAH increased continuously from 27% in 2010 to 42% in 2015 [50]. Nevertheless, targeted PAH drugs are prescribed less aggressively in patients over 65 years of age, which may impair survival, even after adjusting for age, when compared with younger PAH patients [51]. Our study (including a large spectrum of PAH patients) indicates a late initiation of combination therapy in patients over 65 years of age. This is comparable to recently published data from the COMPERA registry [46], in which the proportion of older patients receiving combination PAH therapy also increased over time. In our study, “typical” PAH patients received early combination therapy, as suggested by the guidelines, while older patients with more risk factors and comorbidities received this form of therapy later.

Despite these differences, the outcome of patients remaining on monotherapy during the entire observation period was not different from that of patients receiving dual or triple therapy. This was true for “atypical” as well as for “typical” PAH patients, although the number of patients was too small for a reliable survival analysis within each of these groups.

## Conclusion

Considering these results, upfront combination therapy for “atypical” PAH patients may not be needed when PAH is complicated by advanced age and multiple comorbidities, since the outcome of monotherapy in these patients appears to be comparable to that of dual or triple therapy in “typical” PAH patients.

## Data Availability

To obtain access to the raw data please contact our statistician Dr. Anne Obst, E-Mail: anne.obst@uni-greifswald.de.

## Abbreviations

PAH: pulmonary arterial hypertension
IPAH: idiopathic pulmonary arterial hypertension
PH: pulmonary hypertension
CTEPH: chronic thromboembolic pulmonary hypertension
BMI: body mass index
FC: functional class
COPD: chronic obstructive pulmonary disease
